# Association between GSTM1 and GSTT1 Allelic Variants and Head and Neck Squamous Cell Cancinoma

**DOI:** 10.1371/journal.pone.0047579

**Published:** 2012-10-15

**Authors:** Yang Zhang, Yuanyuan Ni, Hao Zhang, Yongchu Pan, Junqing Ma, Lin Wang

**Affiliations:** Institute of Stomatology, Nanjing Medical University, Nanjing, Jiangsu, China; University of Tennessee, United States of America

## Abstract

**Backgrounds:**

*GSTM1* and *GSTT1* are involved in the detoxification of carcinogens such as smoking by-products, and polymorphisms in these two genes with a result of loss of enzyme activity may increase risk of carcinogenesis. Although many epidemiological studies have investigated the association between *GSTM1* or *GSTT1* null genotype and head and neck squamous cell carcinoma (HNSCC), the results remain conflicting. To elucidate the overall association of *GSTM1*, *GSTT1* and HNSCC, we included all available studies and performed this meta-analysis.

**Methodology/Principal Findings:**

A dataset including 42 articles for *GSTM1*, 32 articles for *GSTT1*, and 15 articles for *GSTM1* and *GSTT1* in combination were identified by a search in PubMed. Associations beween HNSCC and polymorphisms of *GSTM1* and *GSTT1* alone and in combination were analysed by software RevMan 5.1. Stratification analysis on ethnicity and smoking status, sensitivity analysis, heterogeneity among studies and their publication bias were also tested. Association was found in overall analysis between HNSCC and *GSTM1* and *GSTT1* null genotype. Stratified by ethnicity, we found increased risks of HNSCC in carriers with *GSTM1* null genotype in Asian, *GSTT1* null genotype in South American, and dual null genotype in European and Asian. When stratified by smoking, a more significant association of *GSTM1* null genotype with HNSCC risk was observed in smokers.

**Conclusions/Significance:**

This meta-analysis presented additional evidence of the association between *GSTM1* and *GSTT1* polymorphisms and HNSCC risk.

## Introduction

Head and neck neoplasms are the sixth leading cause of death by cancer [1]. The most common histological type is the squamous cell carcinoma, accounting for about 90% of all cases [2], [3]. Being a multifactorial disease, the etiology of head and neck squamous cell carcinoma (HNSCC) is still a much debated question. Smoking of cigarettes, consumption of alcohol and genetic causes are some of the foci of former etiological studies.

Enzymes of the glutathione S-transferase (GST) family are present in eukaryotes and in prokaryotes, which are composed of many cytosolic, mitochondrial, and microsomal proteins. They catalyze various reactions and participate in the phase II biotransformation of xenobiotics. GSTs contribute to the detoxification of by-products of smoking and alcohol and other exogenous chemical carcinogens which may induce HNSCC, so they have been considered as potential candidates for HNSCC susceptibility. Classes*ι* and *μ* of the GST superfamily have been paid lots of attention, which are encoded by *GSTT1* and *GSTM1* genes. The *GSTM1* and *GSTT1* gene have been localized to chromosome 1p13.3 and 22q11.2. Both of the genes are polymorphic and frequent homozygous deletions of the genes presenting null genotype are associated with loss of the corresponding enzyme activity. Therefore, carriers with null genotype will increase the risk of the development of HNSCC due to the decreased ability to detoxify carcinogens theoretically.

In 2003, a meta-analysis conducted by Hashibe *et al.* indicated modest associations of *GSTM1* and *GSTT1* genotypes with head and neck cancer risk [4]. However, more than twenty independent studies from various populations have further examined the relationships between these two genes and HNSCC risk, and still reported conflicting results. Some studies in HNSCC have indicated that the null genotype of *GSTM1* or *GSTT1* is a risk factor of HNSCC development [5]–[7]. However, such an association was not observed in some other groups [8]–[10]. Therefore, it is necessary to reevaluate the association of *GSTM1* or *GSTT1* null genotype with the risk of HNSCC by pooling the new published studies using meta-analysis. The present study included all eligible published case-control studies to establish a relatively comprehensive picture of the relationship between these two genes and HNSCC.

## Materials and Methods

### Selection criteria and identification of eligible studies

Candidate studies were identified through computer-aided literature searches in PubMed for relevant articles in English and Chinese (1995 to May 2012). To identify all articles that studied the association of *GSTT1* and *GSTM1* polymorphisms with HNSCC, we conducted the search using the following keywords and subject terms: ‘GSTT1’ or ‘GSTM1’, and ‘squamous’. We also searched the references cited in the articles and included published works. Abstracts, case-only articles, editorials, review articles and repeated literatures were excluded. Of the articles with the overlapping data, we only included the publication with the most extensive information. The inclusion criteria in the current meta-analysis were as follows: (a) they are unrelated studies; (b) identification of squamous cell carcinoma was histologically confirmed; and (c) they have original data of genotype frequency and provided sufficient information to calculate the odds ratio (OR) or P-value.

### Data extraction

**Figure 1:**
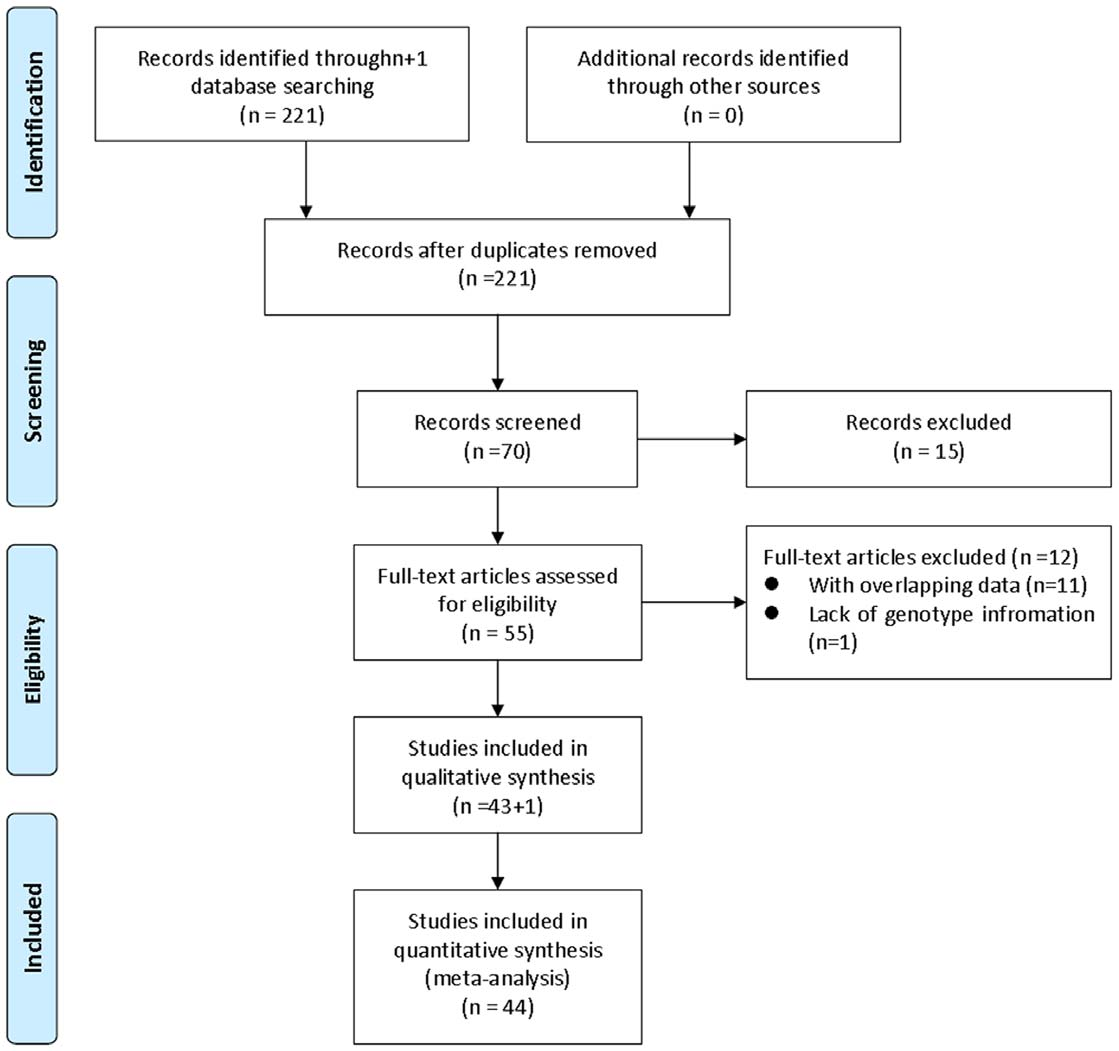
Flow diagram of study identification.

Two reviewers (Zhang Y and Ni Y) independently examined the studies for inclusion in the meta-analysis and collected data on the genotype of *GSTT1* and *GSTM1*. We extracted the following information from each study: first author, year of publication, country, ethnicity, numbers of case and control, smoking status and genotyping information. Disagreements between two reviewers were discussed and resolved with consensus. When essential information was not found in articles, we made effort to get the data from the authors (Figure 1).

### Statistical analysis

The meta-analysis for *GSTM1* or *GSTT1* null genotype or dual null genotype compared HNSCC vs. controls. Odds ratio (OR) and its 95% confidence interval (CI) were assessed for each study. The Cochran's Q-statistic was used to test heterogeneity, and the heterogeneity was considered statistically significant when *P*<0.1 [11]. The Mantel-Haenszel method was used to calculate the OR for the included data in a fixed effects model in the absence of between-study heterogeneity, while random effects model was used for those with heterogeneity. P-value<0.05 was considered statistically significant, and 0.05≤P-value<0.10 was indicated suggestive. In addition, we also performed stratification analyses on ethnicity, smoking and combined analyses of *GSTM1* and *GSTT1* on HNSCC risk. The sensitivity analysis was carried out to test the stability of the pooled effect after excluding individual studies. Begg's funnel plot was used to evaluate publication bias. All above statistical analysis was carried out using the software packages Review Manager (RevMan) 5.1.

## Results

### Eligible studies and meta-analysis databases

We identified 221 articles through the initial computerized search of published work. After reading titles, abstracts, 55 articles were retained. For the analysis of *GSTM1* or *GSTT1*, after discarding 11 articles [7], [12]–[21] due to the overlapping data and 1 article [22] due to lack of essential genotype information, 44 case-control studies [5], [6], [8]–[10], [23]–[60] finally met our criteria for inclusion. Among them, 42 studies described the association between *GSTM1* null genotype and HNSCC, and 32 between *GSTT1* null genotype and HNSCC. For the association between dual null genotype and HNSCC, 1 discarded article [13] containing the distribution information of dual null genotype was reincorporated, and 15 studies were included (Table 1). For the analyses stratified by smoking, eight studies [5], [8], [29], [33], [45], [47], [48], [55] for *GSTM1*, and seven studies [5], [33], [38], [45]–[48] for *GSTT1* were included.

**Table 1:** Characteristics of studies included in meta-analysis.

Author (Ref)	Year	Country	Ethnicity	Case	Control	Whether has genotype distribution information			
						*GSTM1*	*GSTT1*	Dual genes	Tobaccoconsumption
Jahnke et al. (23)	1996	Germany	European	269	216	Yes	Yes	No	No
Park et al. (24)	1997	USA	European	133	133	Yes	No	No	No
González et al. (25)	1998	Spain	European	75	200	Yes	No	No	No
Oude Ophuis et al. (13)	1998	Netherlands	European	185	207	Discarded	Discarded	Yes	No
Cheng et al. (26)	1999	USA	European	162	315	Yes	Yes	Yes	No
Katoh et al. (27)	1999	Japan	Asian	92	147	Yes	Yes	No	No
Morita et al. (28)	1999	Japan	Asian	145	164	Yes	No	No	No
Nazar-Stewart et al. (29)	1999	USA	European	48	144	Yes	No	No	Yes
Sato et al. (30)	1999	Japan	Asian	142	142	Yes	No	No	No
Tanimoto et al. (31)	1999	Japan	Asian	100	100	Yes	No	No	No
Hamel et al. (32)	2000	Canada	European	90	90	Yes	Yes	No	No
Olshan et al. (33)	2000	USA	European	182	202	Yes	Yes	No	Yes
Hahn et al. (34)	2002	Germany	European	94	92	Yes	No	No	No
To-Figureras et al. (35)	2002	Spain	European	204	203	Yes	Yes	No	No
Gronau et al. (36)	2003	Germany	European	187	139	Yes	Yes	Yes	No
Drummond et al. (37)	2004	Brazil	South American	70	82	Yes	No	No	No
Evans et al. (38)	2004	USA	European	283	208	Yes	Yes	No	Yes
Li et al. (39)	2004	China	Asian	89	164	Yes	No	No	No
Drummond et al. (40)	2005	Brazil	South American	87	81	No	Yes	No	No
Gajecka et al. (41)	2005	Poland	European	292	321	Yes	Yes	No	No
Acar et al. (42)	2006	Turkey	Asian	110	197	Yes	Yes	No	No
Biselli et al. (10)	2006	Brazil	South American	60	60	Yes	Yes	Yes	No
Gatta's et al. (43)	2006	Brazil	South American	103	102	Yes	Yes	Yes	No
Oude Ophuis et al. (44)	2006	Netherlands	European	185	285	Yes	Yes	No	No
Peters et al. (45)	2006	USA	European	692	753	Yes	Yes	No	Yes
Sharma et al. (46)	2006	India	Asian	40	87	Yes	Yes	No	Yes
Sugimura et al. (47)	2006	Japan	Asian	122	241	Yes	Yes	No	Yes
Anatharaman et al. (48)	2007	India	Asian	451	727	Yes	Yes	No	Yes
Cha et al. (49)	2007	Korea	Asian	72	209	Yes	No	No	Yes
Suzen et al. (8)	2007	Turkey	Asian	98	120	Yes	Yes	Yes	Yes
Boccia et al. (9)	2008	Italy	European	210	245	Yes	Yes	No	No
Buch et al. (50)	2008	USA	European	196	414	Yes	Yes	No	No
Harth et al. (51)	2008	Germany	European	312	300	Yes	Yes	No	No
Hatagima et al. (52)	2008	Brazil	South American	231	212	Yes	Yes	No	No
Losi-Guembarovski et al. (53)	2008	Brazil	South American	91	81	Yes	Yes	No	No
Amtha et al. (54)	2009	Indonesia	Asian	81	162	Yes	Yes	No	No
Li et al. (55)	2009	China	Asian	76	76	Yes	Yes	No	Yes
Chatzimichalis et al. (56)	2010	Greek	European	88	102	Yes	Yes	No	No
Leme et al. (57)	2010	Brazil	South American	100	100	Yes	Yes	Yes	No
Sam et al. (58)	2010	India	Asian	408	220	Yes	Yes	Yes	No
Soucek et al. (59)	2010	Czech	European	122	179	Yes	Yes	No	No
Lourenço et al. (6)	2011	Brazil	South American	142	142	Yes	Yes	No	No
Ruwali et al. (5)	2011	India	Asian	500	500	Yes	Yes	Yes	Yes
Shukla et al. (60)	2012	India	Asian	150	141	Yes	No	No	No

### Heterogeneity result

Cochran's Q tests indicated heterogeneity exist in different studies in the analysis except studies of dual genes in South American (*P* = 0.51, I^2^ = 0%) and *GSTM1* in non-smokers (*P* = 0.65, I^2^ = 0%). The random or fixed effect model was selected for comparisons with or without heterogeneity, respectively.

### Meta-analysis results

A total of 7584 HNSCC cases and 8576 controls for *GSTM1*, 6255 cases and 7138 controls for *GSTT1*, 2657 cases and 3092 controls for dual genes were investigated.

**Figure 2:**
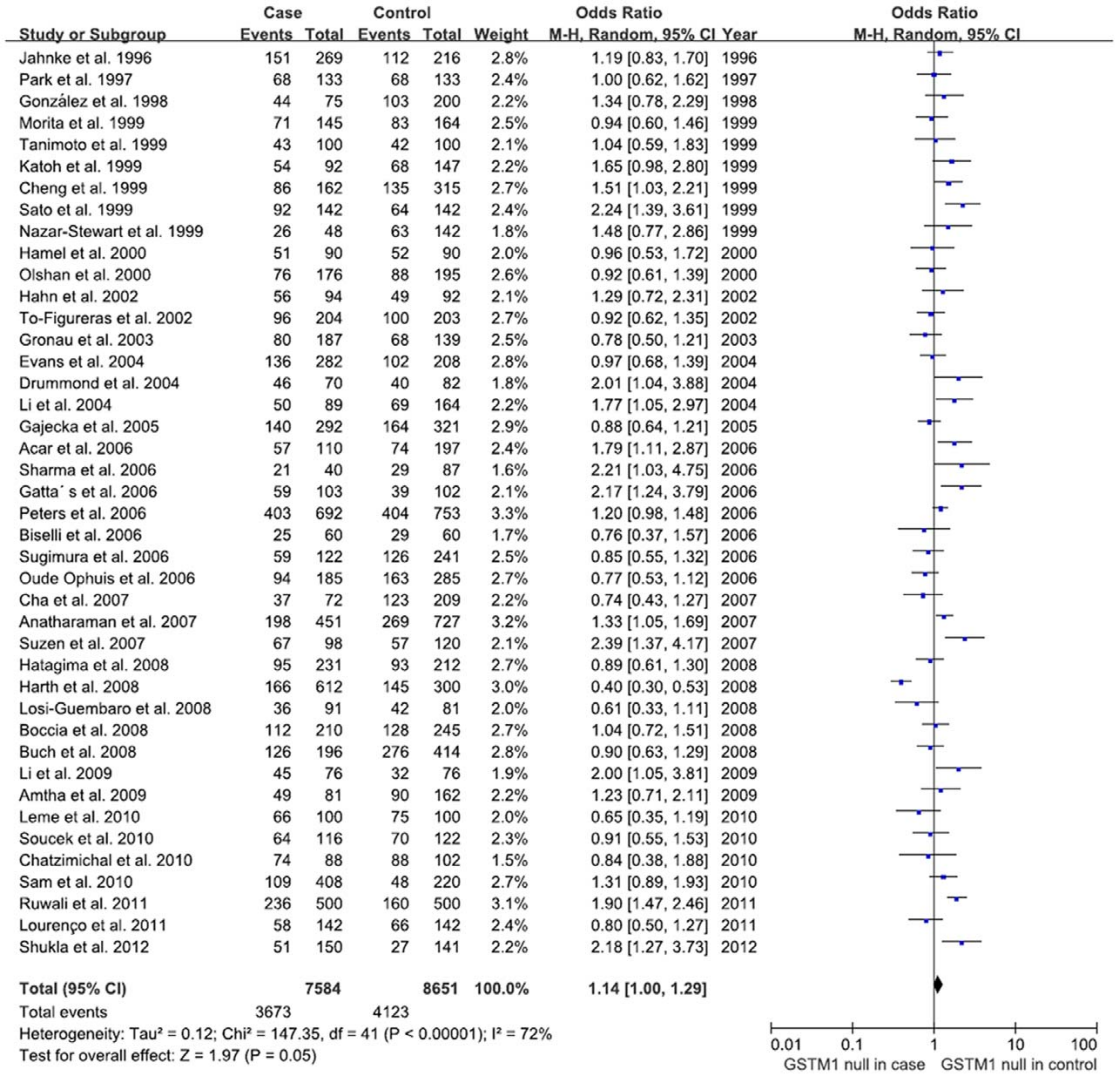
Forest plot of *GSTM1* associated with HNSCC under random-effects model. Each study is shown by point estimate of OR and 95% CI by a horizontal line. The diamond shows the overall risk and the line represent the 95% CI for each meta-analysis. Events: null genotype.

For *GSTM1* polymorphism, the overall meta-analysis showed a suggestively increased risk in null genotype as compared to wild genotype (OR = 1.145, 95% CI: 1.00–1.29, *P* = 0.05) (Figure 2). In sensitivity analysis by temporarily excluding individual studies, no single study substantially affected the pooled OR, indicating that the results of these meta-analyses are stable. Analysis after stratification by ethnicity indicated *GSTM1* null genotype tended to be associated with HNSCC in Asian (OR = 1.48, 95% CI: 1.24–1.75, *P*<0.01), while no significant association was found in European or South American (Table 2).

**Table 2:** Genotype distribution of *GSTM1* and *GSTT1* in different Ethnicities.

	*GSTM1*			*GSTT1*			*GSTM1*+*GSTT1*		
	European	Asian	South Am	European	Asian	South Am	European	Asian	South Am
Cases (n/N^a^)	2049/4111	1239/2676	385/797	766/3458	497/1983	280/814	83/534	200/1769	48/354
Controls (n/N)	2378/4475	1361/3397	384/779	849/3884	586/2476	205/778	57/661	185/2088	48/343
OR^b^	0.96	1.48	1.05	1.21	1.32	1.63	2.01	1.56	0.96
95% CI^c^	0.82–1.13	1.24–1.75	0.71–1.57	0.87–1.69	0.93–1.88	1.03–2.58	1.15–3.53	1.05–2.33	0.62–1.48
*P* ^d^	0.64	<0.00001**	0.80	0.26	0.12	0.04^*^	0.01^*^	0.03^*^	0.85

Abbreviations: ^a^, number of carriers with null genotype/ total number; ^b^, odds ratio; ^c^, confidence interval; ^d^, value for heterogeneity; OR, odds ratio; 95% CI, 95% confidence interval.

** P <0.01; ^*^ 0.01≤P<0.05

**Figure 3:**
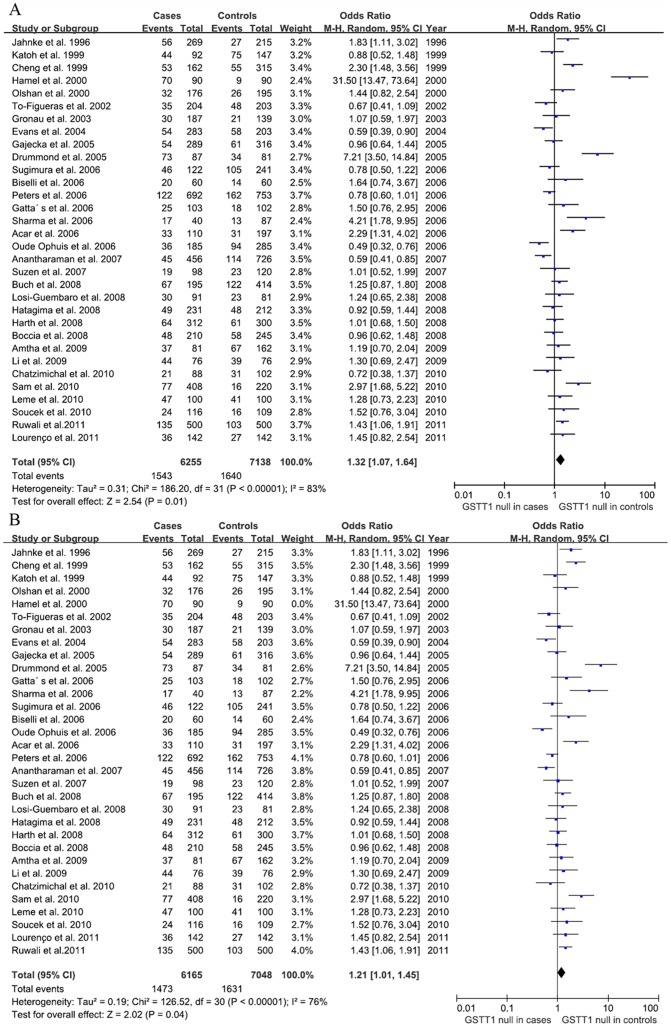
Forest plot of *GSTT1* associated with HNSCC under random-effects model. A: Overall analysis. B: Sensitivity analysis with exclusion of the study by Hamel et al. 2000. The diamond shows the overall risk and the line represent the 95% CI for each meta-analysis. Events: null genotype.

For *GSTT1* polymorphism, null genotype was associated with an increased risk of HNSCC (OR = 1.32, 95% CI: 1.07–1.64, *P* = 0.01) (Figure 3A). Sensitivity analysis showed that the association still exist even with exclusion of the study of Hamel et al. which was obviously deviating from others (OR = 1.21, 95% CI: 1.01–1.45, *P* = 0.04) [32] (Figure 3B). Analysis stratified by ethnicity indicated that *GSTT1* null genotype increased the HNSCC risk in South American (OR = 1.63, 95% CI: 1.03–2.58, *P* = 0.04) (Table 2).

**Figure 4:**
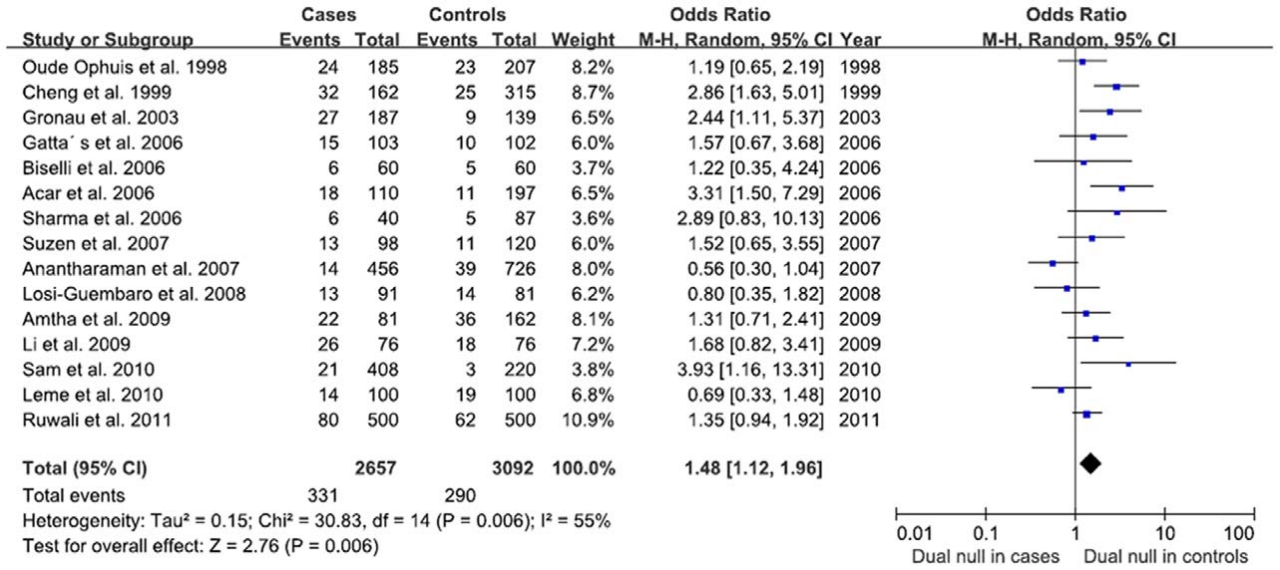
Forest plot of *GSTM1* and *GSTT1* associated with HNSCC under random-effects models. The diamond shows the overall risk and the line represent the 95% CI for each meta-analysis. Events: null genotype.

Combined analysis of *GSTM1* and *GSTT1* on HNSCC risk showed that OR of individuals with dual null genotype was elevated (OR = 1.48, 95% CI: 1.12–1.96, *P* = 0.006) compared to *GSTM1* or *GSTT1* individual null genotype (Figure 4). After stratification for ethnicity, we observed a significant association for HNSCC in European (OR = 2.01, 95% CI: 1.15–3.53, *P* = 0.01) and Asian (OR = 1.56, 95% CI: 1.05–2.33, *P* = 0.03) populations among *GSTM1* and *GSTT1* dual null individuals (Table 2). The exclusion of individual studies did not change these results qualitatively.

We further performed stratification analysis by smoking status. As shown in Table 3, significant association of *GSTM1* deletion with risk of HNSCC was observed in smoking group (OR = 1.51, 95% CI: 1.05–2.17, *P* = 0.03) but not in non-smoking group (OR = 1.14, 95% CI: 0.90–1.43, *P* = 0.28). However, we did not found any significant associations for *GSTT1* in either smokers (OR = 1.01, 95% CI: 0.64–1.60, *P* = 0.96) or non-smokers (OR = 1.13, 95% CI: 0.68–1.86, *P* = 0.64) (Table 3), which may be due to the limited number of study with smoking information.

**Table 3:** Genotype distribution of *GSTM1* and *GSTT1* in different smoking status.

	*GSTM1*		*GSTT1*	
	Non-smoker	Smoker	Non-smoker	Smoker
Cases (n/N^a^)	255/455	862/1638	94/462	347/1671
Controls (n/N)	473/1031	658/1519	222/981	325/1527
OR^b^	1.14	1.51	1.13	1.01
95% CI^c^	0.9–1.43	1.05–2.17	0.68–1.86	0.64–1.6
*P* ^d^	0.28	0.03*	0.64	0.96

Abbreviations: ^a^, number of carriers with null genotype/total number; ^b^, odds ratio; ^c^, confidence interval; ^d^, value for heterogeneity; OR, odds ratio; 95% CI, 95% confidence interval

* 0.01≤P<0.05

### Publication bias

Funnel plots were performed to assess the publication bias, and these shapes did not suggest any obvious evidence of asymmetry in the analyses of *GSTM1*, gene-gene interaction, and *GSTT1* analysis stratified by smoking status. When one study [32] for *GSTT1* analysis and two studies [46], [49] for *GSTM1* analysis stratified by smoking status were omitted, funnel plots illustrated symmetric shape.

## Discussion

Genetic factors play an important role in the etiology of tumors. For HNSCC, genes encoding xenobiotic-metabolizing enzymes (XMEs) are some of the most likely candidates that could affect individual's susceptibility to the disease, due to their involvement of the metabolic activation and detoxification of the environmental carcinogens [61]. Conjugation is one of the most common pathways of xenobiotic metabolism and is considered phase II metabolism which is catalyzed by multiple enzyme superfamilies including Glutathione S-transferases (GSTs). GSTs mediate the reactions of glutathione with electrophiles, resulting in the elimination of potentially carcinogenic chemicals [62]. *GSTM1* and *GSTT1* genes belonging to GSTs have been studied extensively due to their important detoxification function and high-frequency polymorphisms. *GSTM1* and *GSTT1* homozygous deletions (null genotype) may lead to deficient enzyme activity [63]. In the present study, the overall frequency of *GSTM1* and *GSTT1* null genotype in controls were 47.65% and 23.77% respectively in accordance with other studies [64]–[67]. After stratification for ethnicity, the frequency of *GSTM1* and *GSTT1* null genotype in controls in European, Asian and South American were 53.14%, 40.06%, 49.29% and 21.86%, 23.67%, 26.34% respectively, which indicated ethnic differences.

The importance of *GSTM1* and *GSTT1* polymorphisms effects on HNSCC has been a concern in recent years, but the data of existing studies are contradictory. An increase in the risk of HNSCCC was observed in cases with null genotypes of *GSTM1* or *GSTT1* in some studies [5]–[8]. However the risk was not found in other studies. For example, Boccia [9] and Biselli [10] did not find the association between *GSTM1* or *GSTT1* and HNSCC. Although the confused effect of these polymorphisms may be a result of various reasons such as demographic features of subjects and different life styles, comparatively small sample size in individual study might lead to lower statistical power and bias. The present meta-analyses of 42 studies including 7584 cases and 8651 controls for analysis of *GSTM1*, 32 studies including 6255 cases and 7138 controls for analysis of *GSTT1*, and 15 studies including 2657 cases and 3092 controls provide more comprehensive information on the relationships between two genes and HNSCC.

This meta-analysis showed that both *GSTM1* and *GSTT1* null genotype confers susceptibility to HNSCC in the overall analysis. *GSTM1* can deals with large hydrophobic electrophiles including polycyclic aromatic hydrocarbons derived epoxides (PAH) [68], [69], while *GSTT1* targets a more restricted kind of compounds, like monohalomethane and ethylene oxide [70]. Different GST isoforms exhibit overlapping substrate specificity, combinations of *GSTM1* and *GSTT1* null genotype may theoretically confer a higher risk to HNSCC. Comparing to homozygous deletion of *GSTM1* and *GSTT1* alone, deletion of two genes in combination significantly increases the risk of HNSCC as showed in our combined analysis, indicating a synergenic role of *GSTT1* and *GSTM1* in cancergenesis.

Analyses after stratification by ethnicity revealed ethnicity-specific associations between two genes and HNSCC. Our findings indicate that *GSTM1* may be an important factor in Asians in the development of HNSCC, which is similar to the results reported by Hashibe et al. [4]. However, *GSTT1* but not *GSTM1* may be important in South Americans, while *GSTM1* and *GSTT1* in combination play a vital role in Europeans and Asians. This result may be attributed to the different habits of smoking, alcohol consumption, intake of food and different genetic backgrounds in different ethnic groups.

Both *GSTT1* and *GSTM1* can prevent the accumulation of tobacco smoke carcinogens, and compared with non-smokers, mutations of these two genes theoretically increase the risk of HNSCC in smokers. To investigate potential gene-environment interaction, we stratified the data by smoking status. A significant association was observed in smokers with *GSTM1*, whereas no difference was observed between smokers and non-smokers for *GSTT1*. Previous studies showed that *GSTT1* and *GSTM1* are involved in the detoxification of carcinogens such as smoking by-products, and polymorphisms in these two genes with a result of loss of enzyme activity may increase risk of carcinogenesis and have different role in detoxification. [68]–[70]. Although we found higher risk of *GSTM1* null genotype in smokers (OR = 1.51) than non-smokers (OR = 1.14), further individual large study are required to evaluate the interaction of *GSTM1* and smoking on HNSCC risk.

Although our result of this meta-analysis is constructive, its limitations and some potential bias should be addressed. First, despite that a well-designed search strategy was used to identify eligible studies, it was possible that some relevant studies were not included. This study only focused on full-text papers published in English and Chinese in PubMed, so some eligible studies in other languages or in other databases might be missed. Second, adjustments over age, gender and other environmental factors such as alcohol drinking might help better detect the association between *GSTM1*, *GSTT1* and HNSCC. If available detailed individual data are enough for an adjusted estimate in the future, a more precise analysis should be conducted. Third, ethnicity was determined roughly by subject's country due to inadequate available data, and this classification can help us have a regional concept of these genes functions. Fourth, the controls in the included studies were recruited in different ways and not uniformly defined, which may have distorted the meta-analysis. Finally, because all the studies were designed with retrospective studies, we cannot clearly determine the causal relationship between the risk factor and HNSCC. Given the limitations and biases above, the conclusions or interpretations made from the results of this meta-analysis should be explained with caution.

## Conclusions

The results of this meta-analysis suggest that *GSTM1* and *GSTT1* null genotypes may be associated with an increased risk of HNSCC. Further large well-designed studies are warranted to confirm these findings.

## Supporting Information

Prisma Checklist S1 (DOC)
